# microRNA-1 Regulates NCC Migration and Differentiation by Targeting *sec63*

**DOI:** 10.7150/ijbs.35357

**Published:** 2019-09-07

**Authors:** Dongyue Wang, Yajuan Weng, Shuyu Guo, Wenhao Qin, Jieli Ni, Lei Yu, Yuxin Zhang, Qingshun Zhao, Jingjing Ben, Junqing Ma

**Affiliations:** 1 Jiangsu Key Laboratory of Oral Diseases, Nanjing Medical University, Nanjing 210019, China; 2 MOE Key Laboratory of Model Animal for Disease Study, Model Animal Research Center, Nanjing University, Nanjing 210029, China; 3 Department of Pathophysiology, Key laboratory of Cardiovascular Disease and Molecular Intervention, Nanjing Medical University, Nanjing 210029, China

**Keywords:** Cranial Defect, Neural Crest Cells, microRNA, Sec63, iTRAQ

## Abstract

**Background/Aims**: Neural crest cells play a vital role in craniofacial development, microRNA-1 (miR-1) is essential in development and disease of the cardiac and skeletal muscle, the objective of our study is to investigate effects of miR-1 on neural crest cell in the craniofacial development and its molecular mechanism.

**Methods**: We knocked down miR-1 in zebrafish by miR-1 morpholino (MO) microinjection and observed phenotype of neural crest derivatives. We detected neural crest cell migration by time-lapse. Whole-mount in situ hybridization was used to monitor the expressions of genes involved in neural crest cell induction, specification, migration and differentiation. We performed a quantitative proteomics study (iTRAQ) and bioinformatics prediction to identify the targets of miR-1 and validate the relationship between miR-1 and its target gene sec63.

**Results**: We found defects in the tissues derived from neural crest cells: a severely reduced lower jaw and delayed appearance of pigment cells. miR-1 MO injection also disrupted neural crest cell migration. At 24 hours post fertilization (hpf), reduced expression of tfap2a, dlx2, dlx3b, ngn1 and crestin indicated that miR-1 deficiency affected neural crest cell differentiation. iTRAQ and luciferase reporter assay identified SEC63 as a direct target gene of miR-1. The defects of miR-1 deficiency could be reversed, at least in part, by specific suppression of sec63 expression.

**Conclusion**: miR-1 is involved in the regulation of neural crest cell development, and that it acts, at least partially, by targeting sec63 expression.

## Introduction

Malformation caused by craniofacial dysplasia is a common birth defect that seriously affects human health. Most hard and soft craniofacial tissues are derived from the neural crest cells. The neural crest cells, a multipotent cell population, are induced at the neural plate border during neurulation 1. After epithelial-mesenchymal transition, neural crest cells migrate along a specific route. Depending on extrinsic cues, they differentiate into various cell types and form structures and organs such as the nervous system, neuroendocrine cells, smooth muscle cells of the cardiac and pigment cells 2. In the head, neural crest cells migrate from hindbrain rhombomeric regions to the pharyngeal arches where they form the facial cartilage, bone and connective tissue. Three distinguished major streams can be characterized: the mandibular, hyoid, and branchial streams 3. Any effect on neural crest cells during development can lead to craniofacial defects 4-6.

microRNAs (miRNAs) are small, single-stranded noncoding RNAs of 18-25 nucleotides. They regulate gene expression at the posttranscriptional level by base-pairing to partially complementary sites in the 3ʹ-untranslated region (3ʹUTR) of their target genes 7, 8. miRNAs may regulate up to 30% of genes, and genes regulated by miRNAs play an essential role in embryonic development 9. In zebrafish, miR-1 is encoded by dre-miR-1-1 and dre-miR-1-2. The mature sequences of the resulting miRNAs are identical, i.e., 5ʹ-uggaauguaaagaaguauguau-3ʹ. In humans, miR-1 is encoded by hsa-miR-1-1 and hsa-miR-1-2, while their mature sequences are also the same, i.e., 5ʹ-uggaauguaaagaaguauguau-3ʹ. The sequences of mature miR-1 from different species, including zebrafish and humans, are identical. This degree of conservation suggests that they have similar functions in different vertebrates. miR-1 has been reported to show a tissue-specific expression pattern in zebrafish muscle, which is similar to that found in mammals 10.

The muscle-specific expression pattern of miR-1 is essential in cardiac and skeletal muscle development and disease. During cardiac development, miR-1 has been reported to control the balance between cell proliferation and differentiation 11. In addition, miR-1 promotes myogenic differentiation and is involved in cell cycle regulation and migration processes. miR-1 also plays an essential role in Chinese sika deer-derived chondrocytes by directly targeting the 3ʹUTR of insulin-like growth factor 1 and by inhibiting the proliferation of these cartilage cells 12. It has also been reported that miR-1 can inhibit cell proliferation and promote cell differentiation and apoptosis 13,14. miR-1 and miR-133 form clusters on the same chromosomal loci of mouse chromosome 2 and chromosome 18. miR-1 promotes myogenesis by targeting histone deacetylase 4 15. Therefore, miR-1 plays a crucial regulatory role in embryonic development and disease. However, it is likely that many more functions of miR-1 are yet to be identified, and its function during neural crest development and pharyngeal cartilage formation remains unknown.

Based on the known role of miR-1 in embryonic development of vertebrates and the high degree of conservation of miR-1 during evolution, we hypothesized that miR-1 might be involved in embryonic development of neural crest-derived tissues in zebrafish through direct targeting of transcripts of proteins responsible for the differentiation of neural crest-derived tissues.

## Materials and Methods

### Animal maintenance

Wild-type zebrafish and the transgenic reporter strain *Tg (sox10: EGFP)* were raised on a 14 h/10 h light/dark cycle at 28.5 °C in the zebrafish facility of the Model Animal Research Center, Nanjing University. All work was performed with the approval of the Ethics Committee of the Stomatological School of Nanjing Medical University. All procedures were carried out according to the guidelines of the Animal Care Committee of Nanjing Medical University.

### Cell culture

Zebrafish neural crest cells were extracted and screened as described previously 16. Embryos (*sox10: EGFP*) at 11 hpf, 24 hpf, and 72 hpf were anesthetized with tricaine and then decapitated in phosphate-buffered saline (PBS). Embryos were enzymatically dissociated with trypsin-EDTA in PBS. Cells were spun down, washed, resuspended in Dulbecco's modified Eagle's medium (DMEM)-F12 (HyClone, Logan UT USA) containing 10% fetal bovine serum (FBS) and 1% penicillin/streptomycin, and then screened by flow cytometry.

### Microinjection of MOs

The following MO antisense oligonucleotides were purchased from Gene Tools LLC (Philomath, OR, USA): miR-1 MO (5ʹ-ATACATACTTCTTTACATTCCA-3ʹ) 17, sec63 MO (5ʹ-CGTACTGAAACTGCTGTCCGGCCAT-3ʹ) 18, and negative control MO (5ʹ-CCTCTTACCTCAGTTACAATTTATA-3ʹ) 19. One to two-cell embryos were injected with 1 nL of MO.

### Whole-mount in situ hybridization

Probes including *crestin*,* foxd3*,* dlx2*,* dlx3b*,* msxb*,* ngn1*,* tfap2a*,* snai1b* and *sec63* were synthesized using a DIG RNA labeling kit (Roche, Indianapolis IN USA) 20-25. The primers listed below were designed by Primer 5.0 software. The following primers were used (forward/reverse):* crestin* (5ʹ-TGCCCTGGAGACGAAACA-3ʹ/5ʹ-CCCACTTCCGATCTGCTT-3ʹ);

*foxd3* (5ʹ-CAAAGCATGTGTCATCTTG-3ʹ/5ʹ-TGAGAATGTCCGGCTGAT-3ʹ);

*dlx2* (5ʹ-GCCAAAGAAAGTCCG-3ʹ/5ʹ-TGGCTGAAGGTGGG-3ʹ);

*dlx3b* (5ʹ-AGCGTATCCCACCAAGAC-3ʹ/5ʹ-ATGCGTTCAAACAGTCCA-3ʹ);

*msxb* (5ʹ-AAGAAGACTTACCTCCCG-3ʹ/5ʹ-TAAATAGTCCTGGCATCG-3ʹ);

*ngn1* (5ʹ-CTCACAACTACATCTGGGCACT-3ʹ/5ʹ-GAGGGTTTCTTCGGGTCA-3ʹ);

*tfap2a* (5ʹ-GGTCACGGCATTGATACTGG-3ʹ/5ʹ-TCGCCTTGGCTGGAAACT-3ʹ);

*snai1b* (5ʹ-GATGCCACGCTCATTTCTT-3ʹ/5ʹ-GACCCGCACTGGTACTTCTT-3ʹ) and *sec63* (5ʹ-GTACGACGACAGTGGCAACA-3ʹ/5ʹ-TATCGGAGGTGCTCCTCTTC-3ʹ). Whole-mount in situ hybridization was carried out on 4% paraformaldehyde (PFA) fixed zebrafish embryos. The prehybridization and hybridization were performed at 65°C for all riboprobes. In situ hybridization signals were examined with sheep anti-digoxigenin-AP Fab fragments. The color reaction was carried out by chromogen substrates (NBT and BCIP).

### Quantitative polymerase chain reaction (qPCR)

For qPCR, total RNA was isolated from embryos using an RNA extraction kit (Takara Biotechnology, Dalian, China). cDNA was reverse-transcribed using a TAKARA PrimeScript RT reagent kit. The primer for miR-1 was designed according to Wu *et al*. (2016), and snRNA U6 was used as a standard for normalization. qPCR was performed according to the manufacturer's instructions, and fold change was calculated using the 2^-△CT^ method as described previously 26. The following primers were used (forward/reverse): U6 (5ʹ- AAAACAGCAATATGGAGCGC-3ʹ);

dre-miR-1 (5ʹ-CGCGTGGAATGTAAAGAAGTATGTA-3ʹ);

*GAPDH* (5ʹ-ACCACAGTCCATGCCATCAC-3ʹ/5ʹ-TCCACCACCCTGTTGCTGTA-3ʹ; and

*sec63* (5ʹ-CGAGTTCACATCCCACAG-3ʹ/5ʹ-GACAGCACCATCTTCTTCC-3ʹ).

### Alcian blue staining

Zebrafish embryos at 4 days post fertilization (dpf) were fixed in 4% paraformaldehyde in PBS before staining with Alcian blue (Sigma Chemical Co, St. Louis, MO, USA). Embryos were transferred to 30:70 glycerol/1% potassium hydroxide and then to 60:40 glycerol/1% potassium hydroxide before incubation for 2-3 days until they were sufficiently translucent. The key angle and length were measured as described previously 27, 28.

### Time-lapse imaging

*Tg (sox10: EGFP)* embryos were anaesthetized and embedded in 0.8% low melting point agarose (Takara Biotechnology, Dalian, China) 29. Confocal stack pictures of the pharyngeal arch region were taken at the indicated time points using a META Zeiss 810 confocal microscope.

### Isobaric tags for relative and absolute quantitation (iTRAQ)

Each sample for iTRAQ was composed of heads of at least 100 embryos. Embryos were anaesthetized at 24 hpf in 1× PBS on ice and stripped for heads. Total proteins were extracted from the heads of embryos. For iTRAQ labeling, approximately 100 μg of proteins were reduced and alkylated by 10 Mm dithiothreitol and 55 Mm iodoacetamide. And then, each sample was digested and labeled with iTRAQ reagents. The analysis was performed by the State Key Laboratory of Reproductive Medicine at Nanjing Medical University 30.

### Western blot

50 embryos were transferred to sterilized centrifuge tubes at 24 hpf. The total proteins were extracted by the ultrasonic fragmentation method. Western blot analysis was conducted according to the previous publication 31. Primary antibody recognizing Sec63 was purchased from Santa Cruz.

### Luciferase reporter assay

The 3ʹUTR of *sec63*, which contains miR-1 binding site, was amplified by PCR with the following primers: F: 5ʹ-CACAACTCGAGCTGTCCTGCTGTTCCACAAAT-3ʹ and R: 5ʹ-TGAGGATCCTTGAGCCCTATTGCCATCCAC-3ʹ. The 3ʹUTR of *pax7* was amplified with the following primers: F: 5ʹ-ATCGCTCGAGGAACCCGAGGTTTGTACG-3ʹ and R: 5ʹ-CACAACACAAGCGGCCGCATTCAATTAATTCTGTCTTCA-3ʹ. The partial 3ʹUTR fragment was cloned into the pMIR-REPORT Luciferase vector (Promega, Madison, WI, USA). For use as a control, the binding site was mutated and then cloned into the same vector.

HEK293T cells (5 × 10^4^ cells per well) were seeded in 24-well plates and cultured in DMEM/F12 containing 10% FBS (Gibco, Grand Island, NY, USA) overnight. After 24 h, dre-miR-1 mimics or negative control and wild-type or mutated reporter vector were transfected into HEK293T cells (duplex as a control). After 48 h, the cell cultures were split and the luciferase activity was determined according to the manufacturer's protocol (Promega, Madison, WI, USA). The luciferase activity of each type of sample was measured at least three times 32.

### Statistical analysis

Data were presented as the mean ± standard deviation. Experiments were conducted separately at least three times. Student's *t*-test (SPSS 19.0) was used to assess differences between groups. *P* value < 0.05 was considered a significant difference.

## Results

### miR-1 is expressed in zebrafish embryos

miR-1 is expressed in the pharyngeal region in zebrafish at 24 hpf 10. To investigate whether miR-1 is expressed in zebrafish neural crest cells, *sox10*-positive (i.e., EGFP-expressing) cells were extracted and isolated by flow cytometry at 11 hpf, 24 hpf, 48 hpf, and 72 hpf from *Tg (sox10: EGFP)* embryos. qPCR analysis showed that miR-1 was expressed at 12 and 24hpf, and the highest level of expression was observed at 24 hpf, suggesting that miR-1 may play an important role in cranial neural crest cell migration and differentiation (Fig. S1).

### Knockdown of miR-1 affects neural crest derivatives

**Fig 1:**
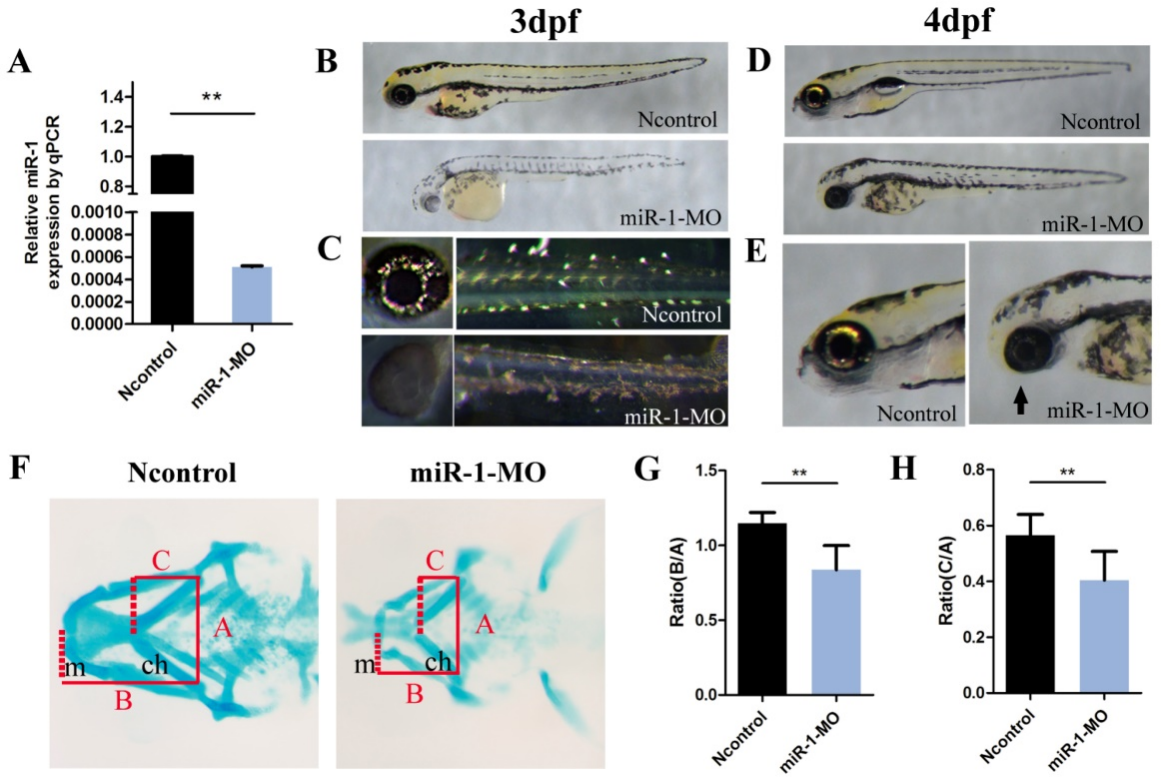
** Knockdown of miR-1 affects neural crest derivatives.** (A) qPCR showed that miR-1 MO knocked down miR-1 efficiently (the experiments were repeated three times). Data are expressed as the mean ± SD, from at least three independent experiments, n=15, **P < 0.01. (B, C) Lateral views of live zebrafish embryos showed pigment cells at 3 dpf. Negative control-injected zebrafish exhibited neural crest-derived pigment cells: black melanophores and iridescent iridophores. The distribution and number of melanophores were reduced in miR-1 morphants. The number of iridophores was reduced in the eye and trunk. (D, E) Lateral views of embryos at 4 dpf. miR-1 morphants displayed a shorter body length with a smaller head and edema around the heart. The lower jaw was reduced in size in the miR-1 morphants (arrows) compared with the negative control embryos. (F) Alcian blue staining showed cartilage development at 4 dpf; ventral views of embryos. (G, H) Ratios B: A and C: A, as shown in (F), in the miR-1 MO-injected and negative MO-injected groups. The definition for lines A, B, and C has been described previously. microRNA-1, miR-1; morpholino, MO; dpf, days post fertilization; mc, Meckel's cartilage; pq, palatoquadrate; ch, ceratohyal cartilage; Data are expressed as the mean ± SD, from at least three independent experiments, n=15, **P < 0.01; N.S., not significant.

Then we used an antisense MO oligonucleotide (miR-1 MO) to inactivate mature miR-1 (Fig. 1A). First, we observed morphological defects induced by miR-1 MO. At 3 dpf, there were fewer melanophores in the yolk sac and in the body of miR-1 morphants than in the negative control MO group (Fig. 1B). There were also fewer iridophores in the eyes and yolk sac extensions in the miR-1 morphants (Fig. 1C). These results indicate that normal expression of miR-1 is required for the development of neural crest-derived chromatophores.

We also found that the mandibular length was reduced and the mouth opening was ventrally rotated (Fig. 1D, E) in the miR-1 MO-injected group. Next, we analyzed the craniofacial morphology at 4 dpf by Alcian blue staining. In miR-1 morphants, the first pair of the cartilaginous pharyngeal arches (mandibular arches) was smaller than in negative controls, and the 3rd to 5th pairs were barely detectable. Simultaneously, the ceratohyal cartilage (second pharyngeal arch) was shifted posteriorly (Fig. F). We measured the positional relationships among joints of the palatoquadrate and hyosymplectic cartilage (Meckel's cartilage) as well as the ceratohyal cartilage by analyzing three lines (Fig. 1F), which have been defined previously 27. The statistical differences of the ratios (B:A and C:A) between the miR-1 MO-injected group and the negative control MO-injected groups are shown in Fig. 1G and H. These results indicate that knockdown of miR-1 expression affected craniofacial chondrogenesis. Therefore, we hypothesized that miR-1 might modulate the induction, migration or differentiation of cranial neural crest cells.

### miR-1 deficiency does not affect neural crest induction and specification, but affects neural crest cell migration and differentiation

In order to determine the stage of neural crest development affected by miR-1 functions, we examined the expression of genes associated with specific stages of neural crest development in miR-1 morphants. A number of genes, including *msxb*, *pax3, dlx3b*, *snail1b*, *foxd3*, *dlx2*, *ngn1*, *crestin* and *tfap2a* have been shown to play important roles in the induction, specification, migration, and differentiation of neural crest cells 33,34.

**Fig 2:**
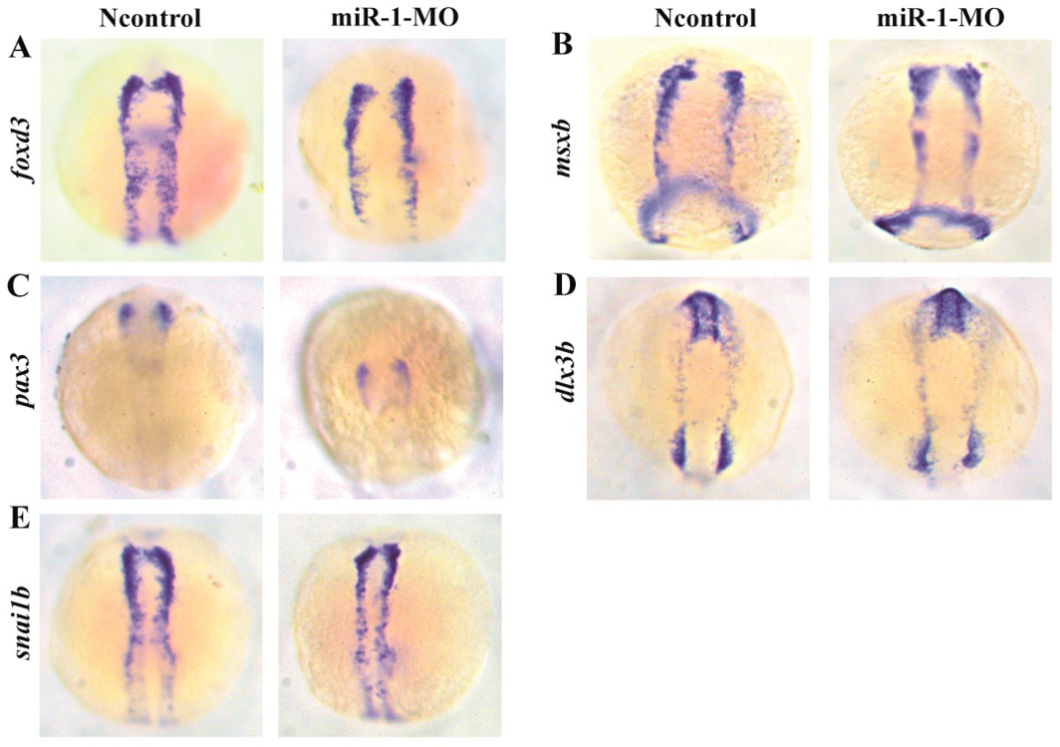
** Expression of selected genes in neural crest induction and specification in miR-1 MO-injected embryos at 11 hpf.** The photographs show a dorsal view with the anterior to the top at 11 hpf. Expression of foxd3 (A), msxb (B), pax3 (C), dlx3b (D), and snai1b (E) in embryos is shown. The expression of foxd3, msxb, pax3, dlx3b and snai1b was similar in negative control embryos and miR-1 morphants. microRNA-1, miR-1; morpholino, MO.

We found that the neural crest specification marker *foxd3* displayed normal expression in miR-1 morphants at 11 hpf (Fig. 2A), approximately at the 3-somite stage (ss) before neural crest cell migration. Expression of the markers *msxb*, *pax3*, *dlx3b*, and *snai1b* in the miR-1 morphants was also normal (Fig. 2B, C, D, E). The normal expression indicated that miR-1 deficiency did not affect the induction and specification of neural crest cells.

**Fig 3:**
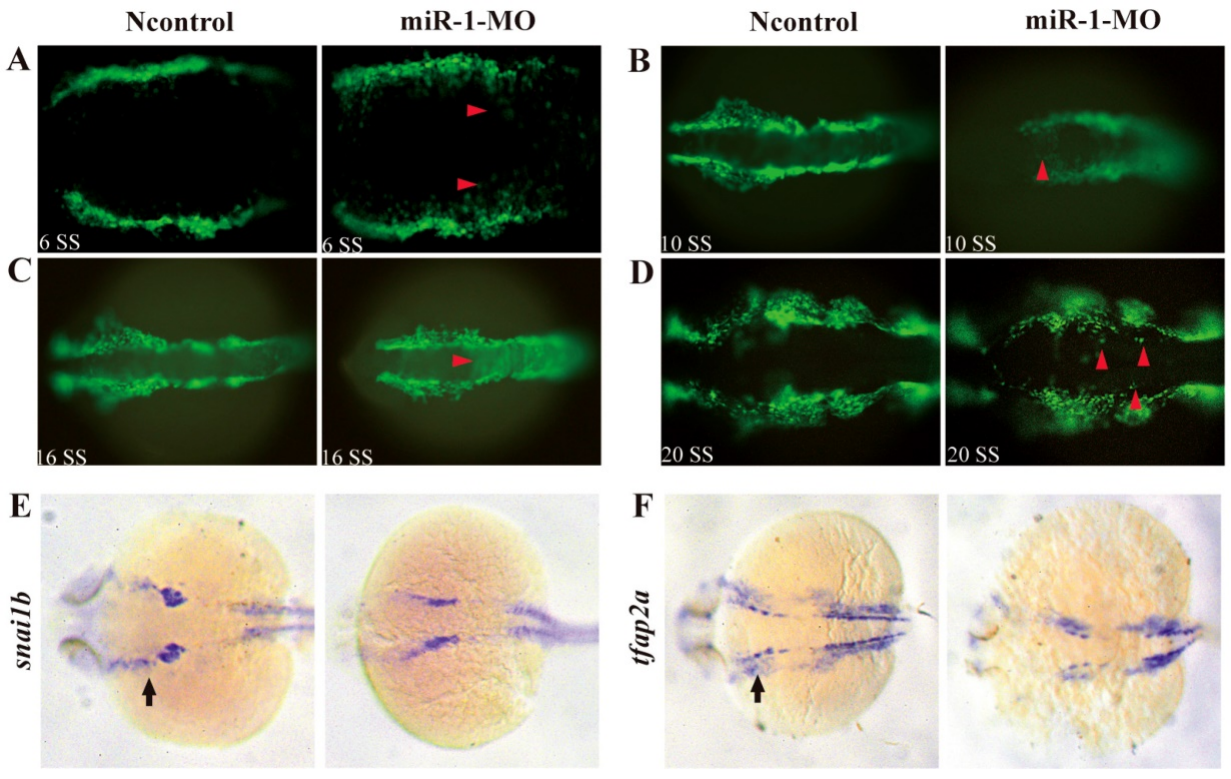
** miR-1 morphants displayed defective migration of neural crest cells.** Live embryo imaging in miR-1 morphants and negative control Tg (sox10: EGFP) embryos was used to analyze the movement of neural crest cells, viewed dorsally. (A-D) Representative images are shown from the time-lapse photography from 6 segment stage (ss) to 20 ss. At 6ss, 10 ss, 16 ss and 20 ss, neural crest cells in miR-1 morphants displayed ectopic EGFP expression (red arrowheads) in the dorsal midline (A, B, C and D). At 24 hpf, expression of snai1b (E) and tfap2a (F) in negative control and miR-1 morphants is shown. At this time, increasing numbers of the migrating lateral line primordium (arrow) expressed tfap2a and snail1b. The expression of neural crest migration markers (snai1b, tfap2a) was reduced (arrows) (E, F). microRNA-1, miR-1; morpholino, MO; ss, segment stage.

Next, we analyzed whether neural crest cell migration was altered in miR-1 morphants. miR-1 MO was injected into *Tg (sox10: EGFP)* embryos. Then, we mapped *sox10* expression during neural crest cell migration at 6 ss, 10 ss, 16 ss and 20 ss. At 6 ss, 10 ss, 16ss and 20 ss, in miR-1 morphants, neural crest cells displayed ectopic EGFP expression in the dorsal midline, while few neural crest cells appeared in the midline in negative control embryos (Fig. 3A, B, C, D). The expression of neural crest migration marker *snai1b* and *tfap2a* was also reduced in neural crest streams in the head (Fig. 3E, F). These data implicated miR-1 regulated neural crest cell migration.

**Fig 4:**
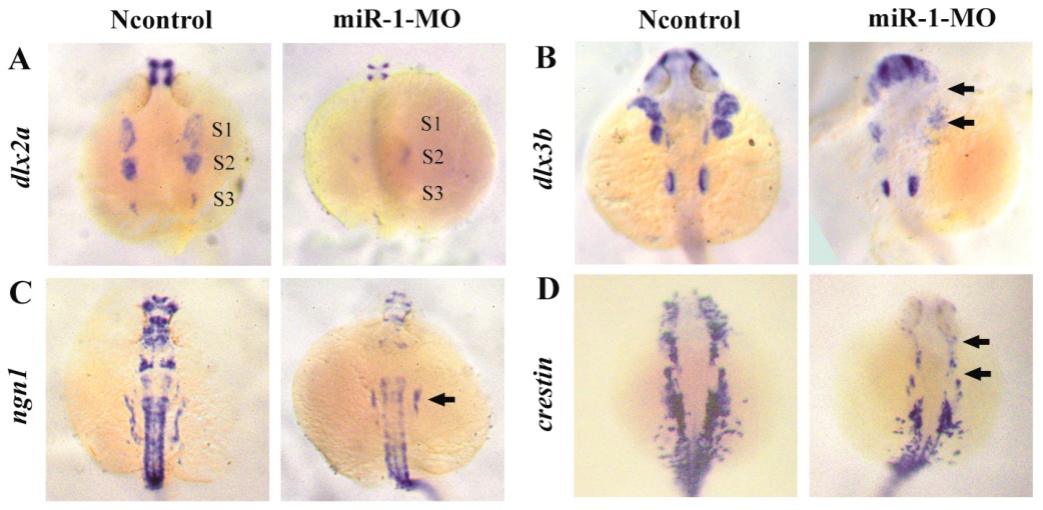
** miR-1 MO-injected embryos displayed defective neural crest cells differentiation.** Embryos were viewed dorsally, anterior to the top, at 24 hpf. Expression of dlx2a (A), dlx3b (B), ngn1 (C), and crestin (D) is shown. Expression of the pharyngeal homing marker gene dlx2a was reduced in the neural crest stream (S1, S2, S3) in miR-1 MO-injected embryos (A). General features of dorso-ventral patterning appeared affected as shown (arrows) by decreased expression of dlx3b (B). ngn1, a marker of neuron differentiation, was downregulated in miR-1 morphants (arrows) (C). The expression of neural crest marker crestin was reduced in the cranial region and the branchial primordia (arrows) (D). microRNA-1, miR-1; morpholino, MO. Taken together, our data suggest that miR-1 loss of function interfered with the development of normal neural crest derivatives through disruption of neural crest cell migration and differentiation.

To determine whether neural crest cell differentiation was altered, we examined the expression of associated genes. *tfap2a* controls the differentiation of cranial neural crest cells into mature chondrocytes. At 24 hpf, *tfap2a* has been reported to be expressed in a highly restricted pattern in the anterior domains 35, 36. We found that, compared with negative control embryos, *tfap2a* gene expression was reduced in miR-1 MO embryos, as can be most notably seen in the pharyngeal arches (Fig. 3F). At 24 hpf in miR-1 MO morphant embryos, the expression of *dlx2a*, a marker for the chondrogenic crest, was clearly reduced in the anterior domain of stream 1 and 2, which would give rise to the mandibular and hyoid arches, and *dlx2a* expression nearly disappeared in the more posterior domain of stream 3, which would migrate into the mandibular arches, the hyoid arches, and the posterior pharyngeal arches (Fig. 4A). Similarly, expression of *dlx3b* in the first and second arches was extremely reduced in the miR-1 MO embryos (Fig. 4B). The gene *ngn1* is a critical marker for dorsal root ganglia development in zebrafish 37, 38. Loss of miR-1 resulted in notable reduction of *ngn1* expression (Fig. 4C). At 24 hpf in negative control embryos, three distinct streams of migrating cranial neural crest cells can be detected by the expression of *crestin* in hindbrain rhombomeres. In miR-1 MO-injected embryos, the expression of *crestin* was nearly absent in the anterior streams and was reduced in the posterior neural crest stream (Fig. 4D). These data implicated miR-1 regulated neural crest cell differentiation.

### miR-1 directly regulates sec63 expression

**Fig 5:**
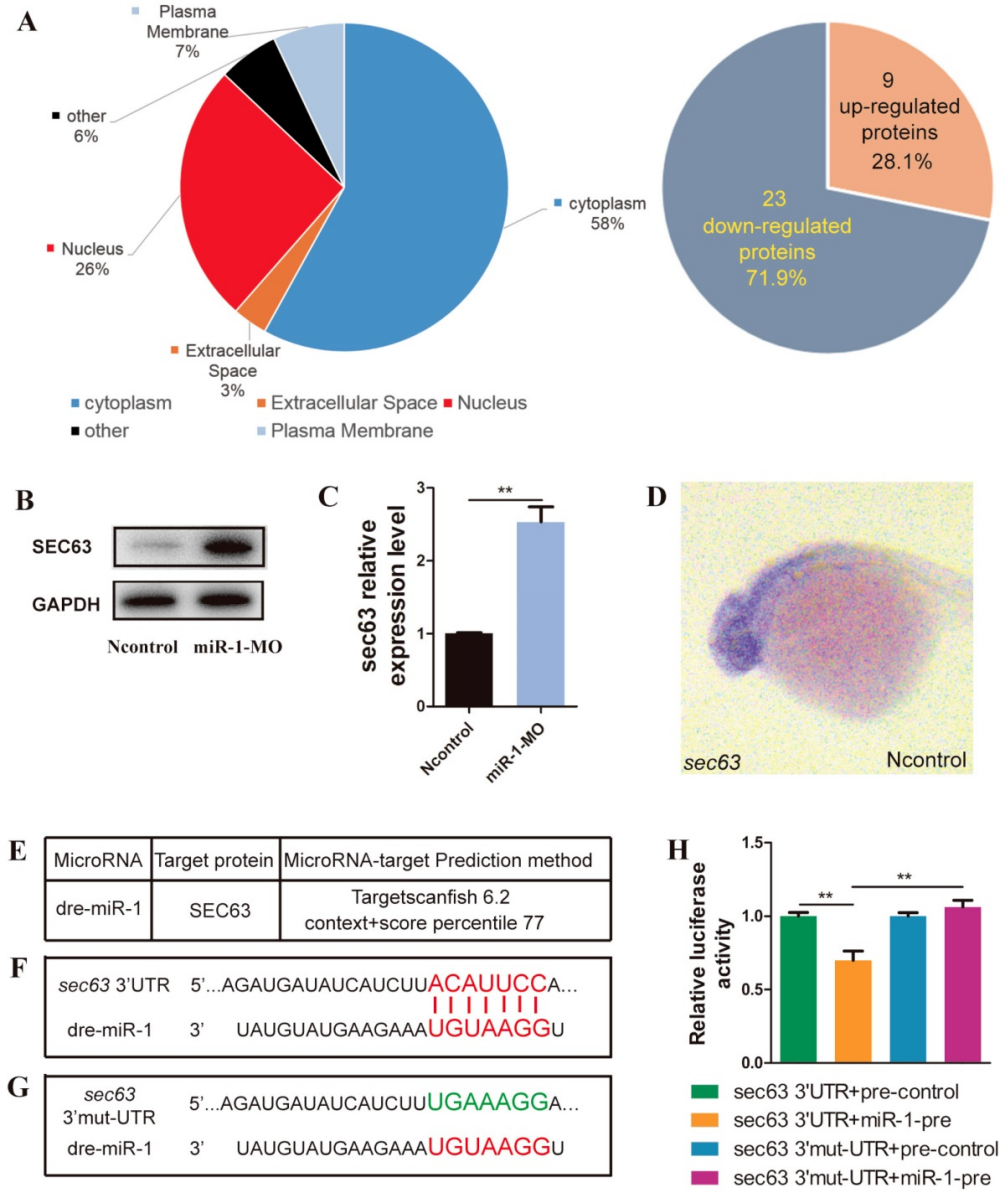
** miR-1 directly targeted sec63.** (A) Subcellular localization of potentially targeted proteins differentially expressed in miR-1 morphants and negative control embryos identified by gene ontology annotations. 9 proteins were upregulated and 23 proteins were downregulated in miR-1 morphants. (B) Protein expression of Sec63 in the control and miR-1 MO-injected groups. (C) A candidate protein for upregulation, sec63, was determined by iTRAQ analysis. Validation of increased sec63 expression in miR-1 morphants by qPCR in vivo at 24 hpf. Data are expressed as the mean ± SD, from at least three independent experiments, n=15, **P < 0.01. (D) Expression of sec63 in embryos at 24 hpf. Embryos were viewed laterally, with the anterior to the left. sec63-3ʹUTR binding site of miR-1 was predicted using TargetScan (E, F). (G) Mutant 3ʹUTR of sec63 in dual luciferase reporter plasmids. Overexpression of miR-1 (miR-1-pre) reduced sec63-3ʹUTR luciferase activity in vitro, but not the luciferase activity of mutated sec63-3ʹUTR. Three independent experiments were performed, and each experiment was carried out in duplicate (H).

We analyzed the differential expression of proteins between miR-1 morphants and negative control embryos by iTRAQ analysis. Compared with negative control embryos, 32 proteins were significantly altered after knockdown of miR-1, including upregulation of 9 proteins and downregulation of 23 proteins (Fig. 5A, Fig. S2). Our results revealed that SEC63 was among the upregulated proteins after miR-1 knockdown, and we chose SEC63 for further analysis because of the reasons below. We found a significant difference in the level of *sec63* Mrna and protein between the miR-1 morphants and negative control embryos at 24 hpf (Fig. 5B, C). We established that the gene *sec63* was expressed in the very domain where the neural crest cells are found (Fig. 5D). Moreover, using the TargetScan search program (Release 6.2), miR-1 was found to be complementary to a RNA sequence from the *sec63* 3ʹUTR. We used a luciferase assay to test the functional interaction between miR-1 and the *sec63* 3ʹ-UTR *in vitro* (Fig. 5E, F, G). The results showed that the miR-1 precursor significantly repressed the expression of luciferase-*sec63*-3ʹUTR but not luciferase-*sec63* 3ʹ-mut-UTR (Fig. 5H). Together, these data indicate that miR-1 directly targeted the 3ʹUTR of *sec63*, thereby modulating the level of *sec63* mRNA and protein.

### Reducing sec63 partly restored the neural crest derivative defects in miR-1 morphants

**Fig 6:**
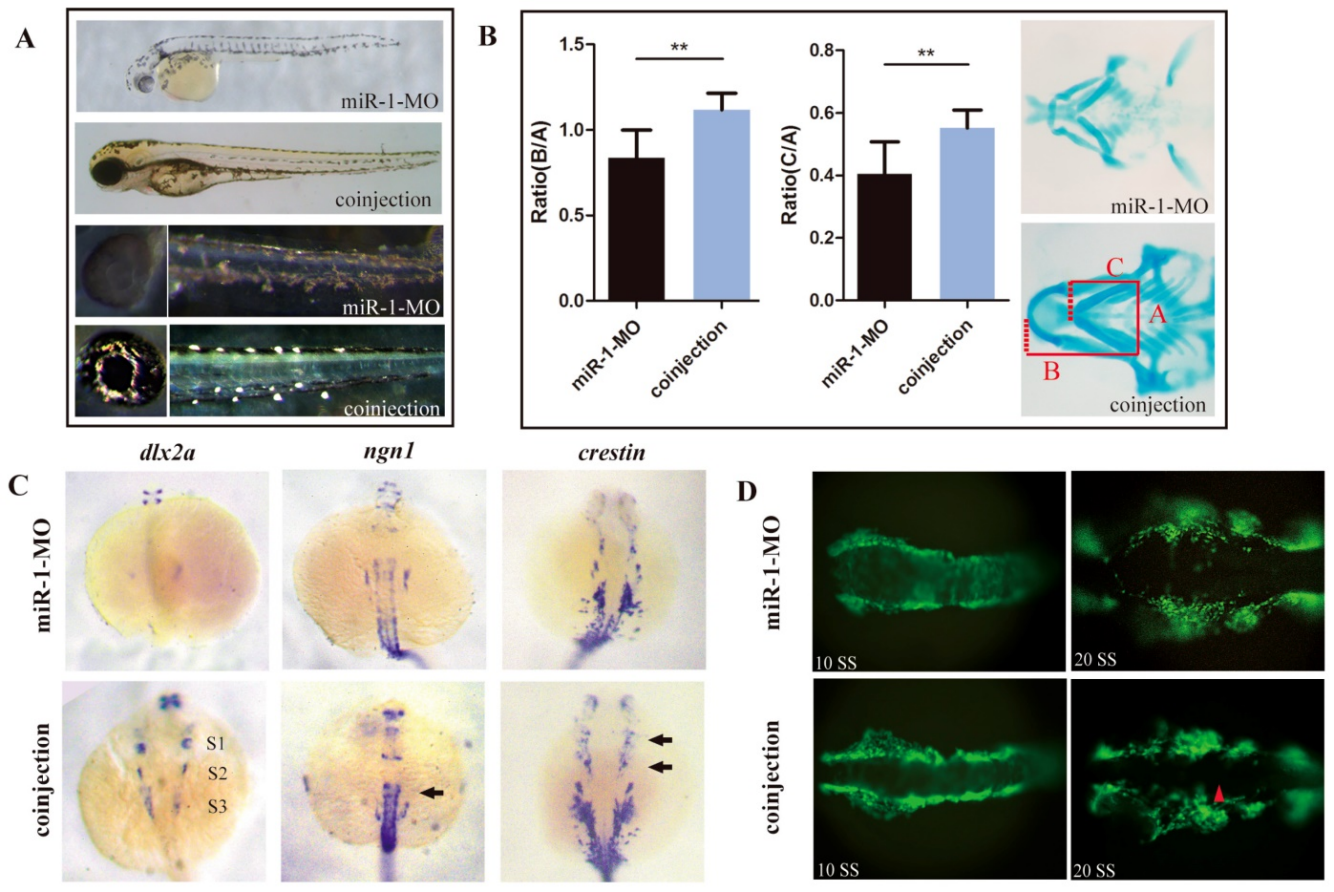
** Reducing sec63 partly restored the neural crest derivative defects in miR-1 morphants.** (A) The defects in melanophores and iridophores were reversed in embryos co-injected with both miR-1 MO and sec63 MO. (B) The craniofacial cartilage were corrected in embryos co-injected with miR-1-MO and sec63-MO. Data are expressed as the mean ± SD, from at least three independent experiments, n=15, **P < 0.01. (C) The expression of dlx2a, ngn1 and crestin were partly rescued at 24 hpf in embryos co-injected with both miR-1 MO and sec63 MO (S1, S2, S3, arrows). (D) The number of ectopic neural crest cells was reduced. microRNA-1, miR-1; morpholino, MO.

We hypothesized that upregulation of *sec63* was one of the likely causes for the defects of neural crest derivatives in miR-1-deficient embryos. To test this possibility, we knocked down *sec63* using a MO oligonucleotide in the miR-1 morphants. Coinjection of miR-1 and *sec63* MOs greatly reversed the delayed appearance of pigment cells (Fig. 6A). Alcian blue staining demonstrated that the defects in craniofacial cartilage were also corrected in coinjected zebrafish at 4 dpf (Fig. 6B). The reduced expression of *dlx2*, *ngn1* and *crestin* was partly corrected at 24 hpf (Fig. 6C). Compared with miR-1 morphants, the number of neural crest cells showing ectopic expression of EGFP during the migration was reduced (Fig. 6D). We concluded that miR-1 regulates neural crest cell migration and derivation in zebrafish by targeting *sec63*.

## Discussion

In this study, we discovered a novel role for miR-1 in neural crest cell s, pharyngeal cartilage, and lower jaw development in zebrafish embryos. Inactivation of miR-1 by antisense MO oligonucleotides led to marked phenotypic differences between miR-1 morphants and negative controls. The majority of the defects in the miR-1 morphants were in cells and tissues derived from the neural crest, including the heart, pigment cells, and craniofacial cartilage, confirming the essential role for miR-1 in neural crest cell development. miR-1 inactivation did not affect neural crest cell induction and specification because there was no major reduction in the number of premigratory neural crest cells at 11 hpf. Neural crest cells displayed ectopic localization in miR-1 morphants at 10 ss and 20 ss, representing early migration and late migration, respectively. At both 10 ss and 16 ss, the movement of cranial neural crest cells was delayed in the miR-1 morphants. Based on our findings, we conclude that the primary role of miR-1 in neural crest cells development is to regulate neural crest cell directional migration and differentiation. In addition, it is possible that miR-1 plays a role in neural crest cell apoptosis and proliferation, but this will require further study.

Several direct targets of miR-1 have been determined *in vivo*, including Hand2 and the Notch ligand *delta* in *Drosophila.* In addition, miR-1 regulates EDN1 in hepatocellular carcinoma progression 11,14,39. Sera 40 predicted that *pax7* might be the direct of miR-1. We carried out luciferase assay to test the functional interaction between miR-1 and the *pax7* 3ʹ-UTR *in vitro* (Fig. S3A). The results revealed that the miR-1 precursor significantly repressed the expression of luciferase-*pax7*-3ʹUTR but not luciferase-*pax7* 3ʹ-mut-UTR (Fig. S3B). However, coinjection of pax7 and miR-1 MOs could not reverse the delayed appearance of pigment cells and the defects in craniofacial cartilage. In order to gain insight into the molecular mechanism of the regulatory effect of miR-1 in neural crest development, we searched different databases for matched sequences between miR-1 and potential target genes in iTRAQ results. After confirmation of function by luciferase assays, we determined zebrafish *sec63* as a direct target of miR-1.

*SEC63* is a member of the endoplasmic reticulum (ER) translocon 41. *sec63* encodes an integral membrane protein of the ER that is highly conserved from yeast to humans. *SEC63* is required in both post-translational and co-translational pathways 42. Mutations in *sec63* cause polycystic liver disease in humans, and even zebrafish *sec63* mutants develop liver pathology. In zebrafish embryos,* sec63* is expressed at all stages examined and in all tissues, and it is broadly expressed in liver and pancreas. Disruption of *sec63* in zebrafish leads to abnormalities in myelinating glia in both the central and peripheral nervous system 43. The function of *sec63* during neural crest development and craniofacial cartilage formation has not been studied yet.

*SEC63* is part of post-translational quality control and processing of polycystin-1 (PC1) and polycystin-2 (PC2) 44. The polycystin complex has been implicated in MAPK/ERK and cAMP pathways 45. Function of the PCs has been found in different cellular and tissue, is their role in regulation of cell migration and tissue morphogenesis 46. When the expression of PCs was changed, it could lead to edema and cardiac defects in embryos 46. And we also observe the edema and cardiac defects in miR-1 MO groups. Then we supposed that *Sec63* was predicted target of miR-1 and responsible for proper neural crest cell migration and differentiation.

To determine whether *sec63* expression is regulated by miR-1, we carried out rescue experiments. Embryos co-injected with both miR-1 MO and *sec63* MO displayed relatively normal body length, head size, heart, and the appearance of pigment cells was also normal. Significantly, the lower jaw extended more anteriorly compared with miR-1 morphant embryos. And the Meckel's cartilage could extend anteriorly beyond (but close to) the eyes. Whole-mount hybridization analyses demonstrated that the expression was similar to that in negative control embryos at 24 hpf. Migration of neural crest cells in miR-1 MO and *sec63* MO morphants was relatively normal. This partial effect of *sec63* MO in miR-1 MO morphants confirms that *sec63* is a target gene of miR-1. These results confirmed the effect of miR-1 on *sec63* that was observed in the iTRAQ experiments. In addition, luciferase reporter assays confirmed that *sec63* is a direct target of miR-1.

Our findings provide the first evidence that miR-1 regulates *sec63* by directly targeting the 3ʹUTR of *sec63*. Taken together, our study suggests a molecular mechanism in which miR-1 participates in the control of *sec63* expression and regulates neural crest migration and formation of Meckel's cartilage during embryonic development in zebrafish. These findings will hopefully help us to better understand craniofacial development and promote effective therapy and treatment for craniofacial malformations.

## Supplementary Material

Supplementary figures.
