# Supplementary material for: microRNA-1 Regulates NCC Migration and Differentiation by Targeting sec63
Source: Int J Biol Sci. 2019 Sep 7;15(12):2538–47. doi: 10.7150/ijbs.35357 (PMC6854364; doi:10.7150/ijbs.35357)
Supplement: Supplementary file 1 — Supplementary figures. [file ijbsv15p2538s1.pdf]

Figure. S1

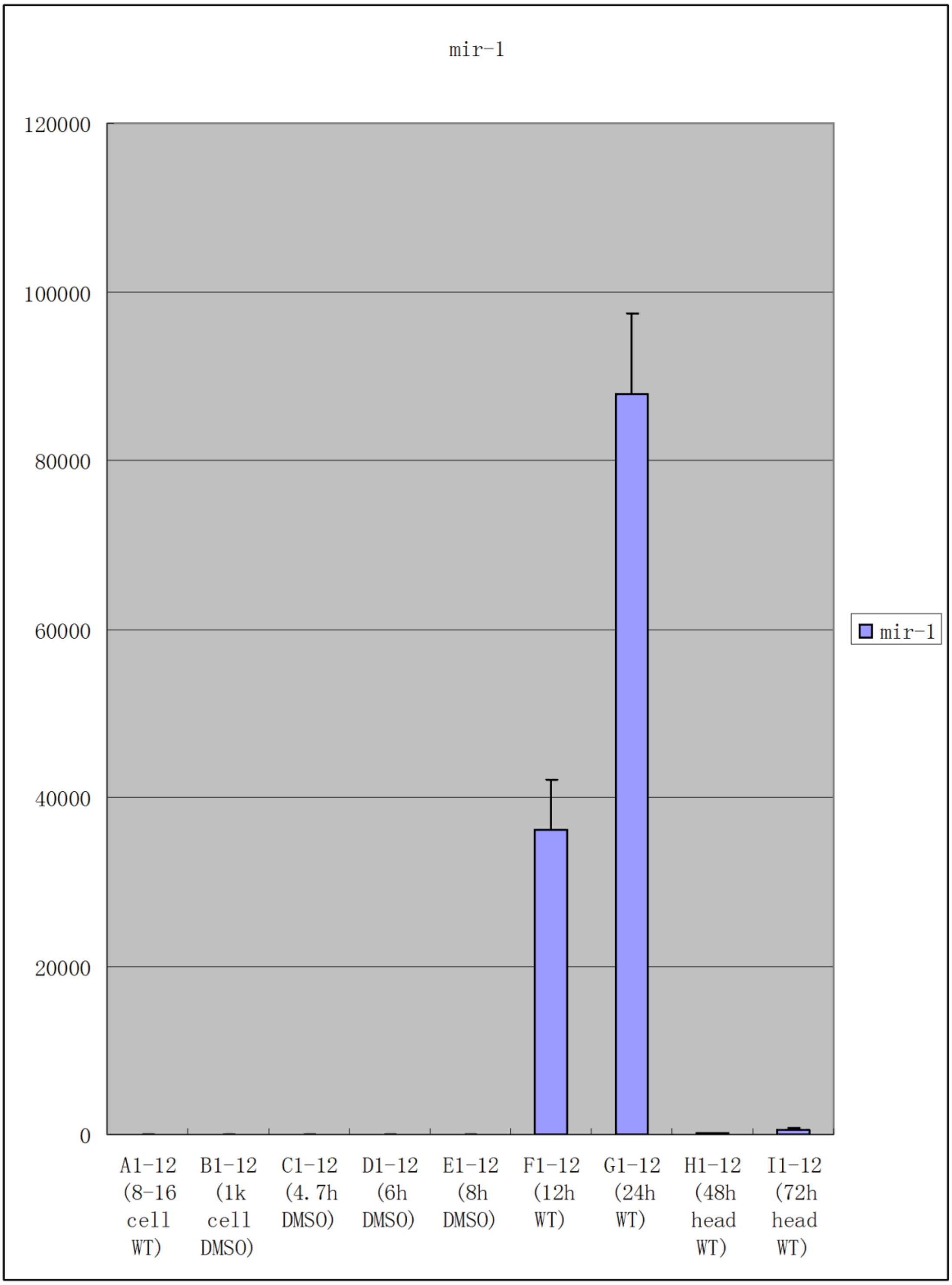

Fig. S1 Temporal pattern of the expression of miR-1 in neural crest cells during embryonic development as determined by qPCR (the experiment was repeated three times). Data are expressed as the mean ± SD n =3, *$P < 0.05$.

Figure. S2

First protein ID	Gene name	Fold change
A0A0G2KP17	wu:fc46h12	-1.407618509
Q566T5	prmt3	-1.316357346
E9QD37	ube2d1a	-1.726948203
D1GJ54	actn1	-1.33995673
I3IT87	ap2m1b	-1.347182078
F1QZ78	gmppab	-1.616065762
B2GQV3	polr2gl	-1.322744528
A7MCK2	hsd17b12b	-1.350172626
Q567D0	zgc:112146	-1.34175085
F1QQZ1	sf3b1	-1.322493586
Q7T312	ccdc25	-1.490204234
F1R9F9	trim33	1.610966599
E7FCM7		1.629989193
Q6IQV0	zgc:86598	-1.380019017
Q6PBM1	glrx5	-1.903557559
F1QYM4	eif4h	-1.48027765
A2AR72	wisp2	-1.759694058
H0WF64	gbe1b	-1.50246823
F1QY34	lrp1ab	-1.334486768
E9QCZ3	si:dkey-28b4.8	1.352511612
Q2TTK0	igf2r	1.520691853
Q803N7	csnk2a1	-1.357966301
F1QBP5	pclob	1.528901407
Q7ZV23	nhp2l1b	1.356070746
F1RB48	mrpl44	-1.415077064
Q5U3T6	pmpca	-1.358033005
Q6DGY6	sec63	1.569462485
X1WDG2	hmgb1b	-1.82869997
Q08CD8	rhebl1	-1.524766591
Q0P478	mrpl20	-1.683154475
B0JZP4	LOC564840	1.428645703
E7FBD5	iqgap1	1.365135089

Fig. S2 The identity of the differentially regulated proteins and their fold regulation.

Figure. S3

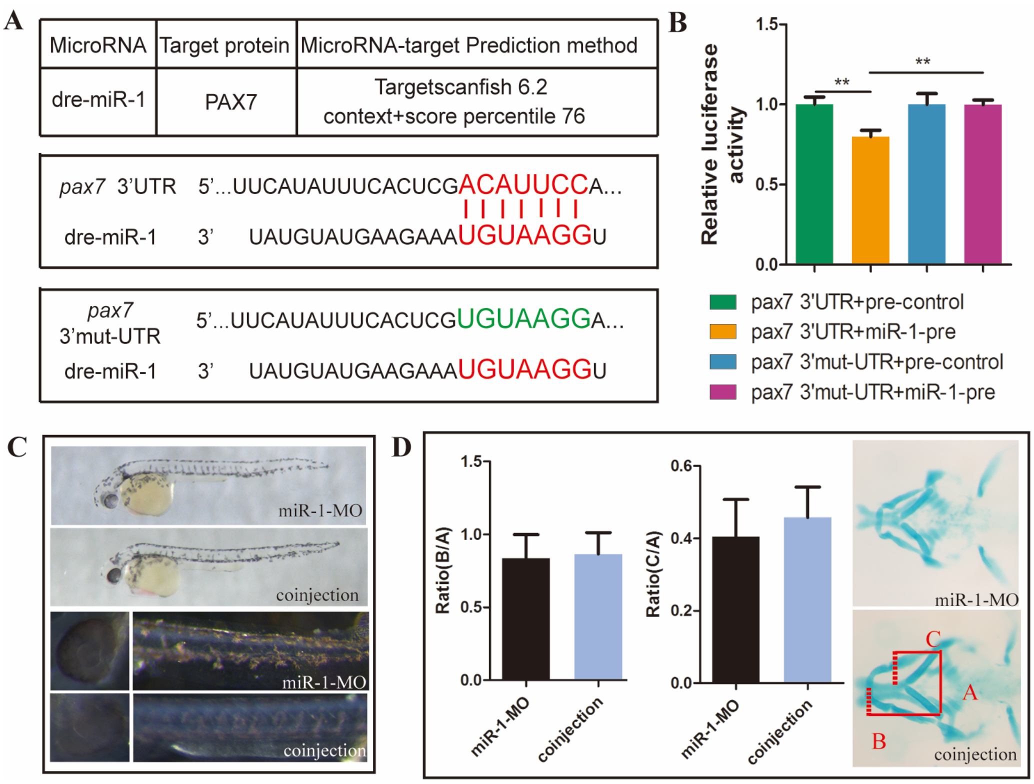

Fig. S3 Pax7-3′UTR had a binding site for miR-1, but the function of miR-1 is indirect by the way of its target Pax7. (A) Pax7-3′UTR binding site of miR-1 was predicted using TargetScan. Mutant 3′UTR of pax7 in dual luciferase reporter plasmids. Overexpression of miR-1 (miR-1-pre) reduced pax7-3′UTR luciferase activity in vitro, but not the luciferase activity of mutated pax7-3′UTR. Three independent experiments were performed, and each experiment was carried out in duplicate (B). (C) The defects in melanophores and iridophores were not reversed in embryos co-injected with both miR-1 MO and pax7 MO. (D) The craniofacial cartilage were not corrected in embryos co-injected with miR-1-MO and pax7-MO. microRNA-1, miR-1; morpholino, MO.
